# Valorization of *Punica granatum* L. Leaves Extracts as a Source of Bioactive Molecules

**DOI:** 10.3390/ph16030342

**Published:** 2023-02-23

**Authors:** Sandra Marcelino, Filipa Mandim, Oludemi Taofiq, Tânia C. S. P. Pires, Tiane C. Finimundy, Miguel A. Prieto, Lillian Barros

**Affiliations:** 1 Centro de Investigação de Montanha (CIMO), Instituto Politécnico de Bragança, Campus de Santa Apolónia, 5300-253 Bragança, Portugal; 2 Laboratório Associado para a Sustentabilidade e Tecnologia em Regiões de Montanha (SusTEC), Instituto Politécnico de Bragança, Campus de Santa Apolónia, 5300-253 Bragança, Portugal; 3 Nutrition and Bromatology Group, Department of Analytical Chemistry and Food Science, Faculty of Science, Universidade de Vigo, 36310 Ourense, Spain

**Keywords:** *Punica granatum* L., phytochemical composition, bioactive properties, biowaste valorization

## Abstract

Due to a lack of innovative valorization strategies, pomegranate processing generates a significant amount of residues with a negative environmental footprint. These by-products are a rich source of bioactive compounds with functional and medicinal benefits. This study reports the valorization of pomegranate leaves as a source of bioactive ingredients using maceration, ultrasound, and microwave-assisted extraction techniques. The phenolic composition of the leaf extracts was analyzed using an HPLC-DAD-ESI/MSn system. The extracts’ antioxidant, antimicrobial, cytotoxic, anti-inflammatory, and skin-beneficial properties were determined using validated in vitro methodologies. The results showed that gallic acid, (-)-epicatechin, and granatin B were the most abundant compounds in the three hydroethanolic extracts (between 0.95 and 1.45, 0.7 and 2.4, and 0.133 and 3.0 mg/g, respectively). The leaf extracts revealed broad-spectrum antimicrobial effects against clinical and food pathogens. They also presented antioxidant potential and cytotoxic effects against all tested cancer cell lines. In addition, tyrosinase activity was also verified. The tested concentrations (50–400 µg/mL) ensured a cellular viability higher than 70% in both keratinocyte and fibroblast skin cell lines. The obtained results indicate that the pomegranate leaves could be used as a low-cost source of value-added functional ingredients for potential nutraceutical and cosmeceutical applications.

## 1. Introduction

Over the last decade, more attention has been paid worldwide to the concept and development of a circular economy model. It is also one of the United Nations’ goals for achieving a more sustainable world by 2030 [1]. In addition, consumers tend to look for products that are more “natural”, “healthier”, “clean-label”, and obtained through ecologically friendly technology [2,3]. Fruit and vegetable biowastes (peels, seeds, shells, pomace, and leaves) are rich sources of bioactive beneficial components. Those compounds contain agricultural, food, cosmetic, and pharmaceutical properties, being a promising way to achieve this goal [4,5,6,7].

One of the oldest known plants is *Punica granatum* L., also denominated by the pomegranate tree. This species is a deciduous shrub in the Lythraceae family and is native to the Mediterranean region. Pomegranate has been shown to provide a wide variety of potential therapeutic properties, such as anti-inflammatory, antioxidant, and cytotoxic activity [8,9,10]. Those properties could be linked to the abundance of phenolic acids, flavonoids, tannins, amino acids, and alkaloids [4,11,12,13,14,15]. Studies have shown the advantages of employing pomegranate peels and seeds as sources of naturally occurring bioactive molecules and functional ingredients, which might be used in the food, pharmaceutical, and other industries [16,17]. Large amounts of non-edible wastes in the form of outer peel, seeds, and pomace are normally discarded, representing more than 40–50% of the whole fruit [18,19,20,21]. Pomegranate leaves, which are also considered non-edible waste, are also one of the plant tissues discarded. They have no applicability and, therefore, generate a large amount of valuable vegetable material. This material could be a rich source of biochemical compounds for several industrial sectors. Flavonols and flavones have already been identified in pomegranate leaves, including catechin, epicatechin, gallocatechin, kaempferol, quercetin, and apigenin [8]. The existence of anthocyanins in early pomegranate leaves further shows that they protect tissues from abiotic and biotic challenges throughout leaf development [22]. The bulk of scholarly papers is focused on the activities of *P. granatum* seeds, flowers, and juice [20,23,24]. In turn, studies regarding the potential therapeutic and functional properties of the pomegranate leaves are scarce [25]. The separation and elimination of the leaves during fruit processing involve a significant capital investment and, as such, the sustainable recovery of bioactive molecules represents an effective strategy to add value to the production chain. The glossy green leaves of *P. granatum* can reach a height of 3 cm [26]. Their bioactive composition, namely secondary metabolites such as phenolic compounds, have been linked to a variety of biological and pharmacological functions [5,21,27,28,29,30,31,32,33]. Properties such as antioxidant, anti-inflammatory, antimetabolic, anticancer, and antibacterial have already been studied [30,33,34].

The present work aims to evaluate the bioactive properties and phenolic composition of pomegranate leaf hydroethanolic extracts obtained using different extraction methodologies, thus contributing to their valorization, and enhancing their possible therapeutic use.

## 2. Results and Discussion

### 2.1. Phenolic Composition

**Figure 1:**
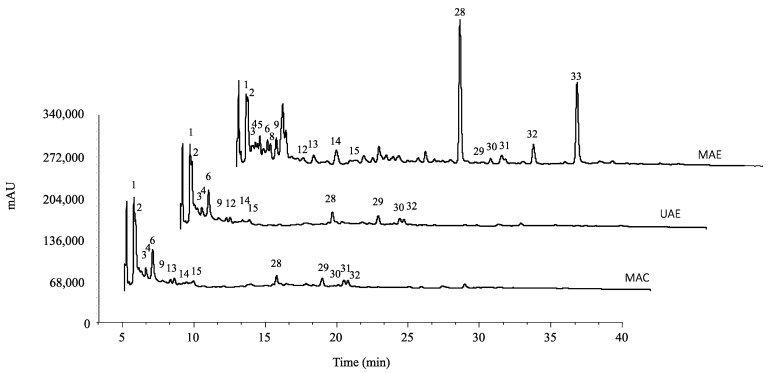
Phenolic profile of *P. granatum* hydroethanolic extracts recorded at 280 nm and their tentatively identified compounds. Peak numbers correspond to the compounds described in Table 1. MAE—microwave-assisted extraction; UAE—ultrasound-assisted extraction; MAC—maceration.

The tentatively identified compounds and their quantification are presented in Table 1 and Table 2, respectively, and the chromatogram is shown in Figure 1. Thirty-three compounds were tentatively identified, thirteen of which were phenolic acids (peaks 1, 2, 3, 4, 5, 6, 8, 9, 10, 12, 13, 14, 15), fifteen flavonoids (peaks 7, 11, 16, 17, 18, 19, 20, 21, 22, 23, 24, 25, 26, 27), and six tannins (peaks 28, 29, 30, 31, 32, 33).

Peak 1 ([M–H]^−^ at *m*/*z* 169) was positively identified as gallic acid based on its retention time, mass, and UV-vis properties in comparison to commercial standards. Peaks 2 and 6 ([M–H]^−^ at *m*/*z* 353) were confirmed as *O*-caffeoylquinic acid, and the base peak provided deprotonated quinic acid (*m*/*z* at 191) and another significant ion belonging to the hydroxycinnamic acid residue at *m*/*z* 179 [caffeic acid-H]^−^. Because of the hierarchical fragmentation pattern outlined by Clifford et al. [35], these assumptions were taken into account. Peak 3 was identified as methyl gallate hexoside ([M–H]^−^ at *m*/*z* 345), presenting a λ_max_ around 278 nm. Díaz-Mula et al. reported a similar compound in their study [36]. Peaks 5 and 8 ([M–H]^−^ at *m*/*z* 355) were identified as glucaric acid (209 *m*/*z*), revealing the loss of a rhamnosyl moiety (- 146 u). Peaks 9 ([M–H]^−^ at *m*/*z* 477), 13 ([M–H]^−^ at *m*/*z* 609), 14 ([M–H]^−^ at *m*/*z* 433), and 15 ([M–H]^−^ at *m*/*z* 447) were tentatively identified as methyl ellagic acid hexoside, ellagic acid (*p*-coumaroyl) hexoside, ellagic acid pentoside, and methyl ellagic acid pentoside, respectively, all revealing a *λ*_max_ around 358 nm and a MS^2^ fragment at *m*/*z* 301, characteristic of ellagic acid. Peak 12 ([M–H]^−^ at *m*/*z* 355) revealed, after the loss of a hexoside unit (−162 u), a product ion equivalent to a ferulic acid molecule (193 *m*/*z*) and was tentatively identified as ferulic acid hexoside.

From all the phenolic acids identified, gallic acid was the compound that presented the highest concentrations through UAE and MAC (Table 2).

Regarding the flavonoids, peaks 7 and 11 ([M–H]^−^ at *m*/*z* 289) and peaks 17 ([M–H]^−^ at *m*/*z* 609) and 19 ([M–H]^−^ at *m*/*z* 463) were identified according to the standard compounds as (+)-catechin, (-)-epicatechin, quercetin-3-*O*-rutinoside, and quercetin-3-*O*-glucoside, respectively. Peaks 20 ([M–H]^−^ at *m*/*z* 477) and 23 ([M–H]^−^ at *m*/*z* 505) were assigned as quercetin-*O*-glucuronide and quercetin-*O*-acetyl-glucoside, respectively. They presented MS^2^ fragments, corresponding to the loss of a glucuronide (- 176 u) and acetyl-glucoside (- 42-162 u). Peaks 16 ([M–H]^−^ at *m*/*z* 491), 24 ([M–H]^−^ at *m*/*z* 447), and 26 ([M–H]^−^ at *m*/*z* 461) corresponded to isorhamnetin derivatives (λ_max_ around 354 nm, and MS^2^ fragment at *m*/*z* 315). Peak 16 presented a pseudomolecular ion [M–H]^−^ at *m*/*z* 491, which released fragments at *m*/*z* 315 ([M–H–176]^−^), representing the loss of a glucuronyl moiety. Peak 24 was characterized as isorhamnetin-*O*-pentoside, and peak 26 was identified as isorhamnetin-*O*-rhamnoside based on its fragmentation pattern. Peaks 18 ([M–H]^−^ at *m*/*z* 593), 21 ([M–H]^−^ at *m*/*z* 447), and 22 ([M–H]^−^ at *m*/*z* 461) were identified as luteolin derivatives. These compounds were tentatively identified as luteolin-7-*O*-rutinoside, luteolin-7-*O*-glucoside, and luteolin-*O*-glucuronide, based on their pseudomolecular ions and MS^2^ fragment losses corresponding to rutinosyl (- 308 u), hexosyl (- 162 u), and glucuronyl (- 176 u) moieties, respectively. Peak 25 (kaempferol-3-*O*-glucoside) was identified by its retention, mass spectra, and UV-vis characteristics by comparison with commercial standards (max. around 334 nm, and MS^2^ fragment at *m*/*z* 285). Luteolin derivatives have also been identified in the pomegranate’s peel [37,38]. Peak 27 was assigned to a flavone, apigenin-*O*-glucuronide ([M–H]^−^ at *m*/*z* 445), releasing an MS^2^ fragment at *m*/*z* 269 ([M–H–176]^−^), apigenin with a loss of a glucuronyl moiety.

Epicatechin (peak 11) was the major flavonoid compound found in MAE and MAC extract, while the UAE revealed quercetin-*O*-glucuronide as the main compound (Table 2).

Finally, peaks 28–33 were assigned as ellagitannins. Peak 29 ([M–H]^−^ at *m*/*z* 1083) was identified as punicalagin, which is very characteristic of *P. granatum*, and this peak was also described by Fischer et al. [39] and Lu et al. [40]. Similarly, peak 28 ([M–H]^−^ at *m*/*z* 951) was identified as granatin B (galloyl-HHDP-DHHDP-hexoside), which was previously identified by Canuti et al. [41]. Peak 30 ([M–H]^−^ at *m*/*z* 799) was identified as lagerstannin A, which has also been reported in *P. granatum* [39]. The mass spectral characteristics of peak 31 ([M–H]^−^ a *m*/*z* 785, fragments *m*/*z* 633 and 301) coincide with digalloyl-HHDP-glucose, also described in pomegranate epicarp [42]. Peaks 32 ([M–H]^−^ at *m*/*z* 783) and 33 ([M–H]^−^ at *m*/*z* 935) were tentatively identified as pedunculagin (bis-HHDP-glucose) and casuarictin (1-β-*O*-galloyl-pedunculagin), respectively, and previously reported by Singh et al. [43]. In the latter case, the gallic acid would not be bound to punicalagin by the carboxyl group, as denoted by the fragment at *m*/*z* 783 corresponding to the loss of gallic acid itself (−152 u).

Granatin B (peak 28) was the hydrosoluble tannin found in higher concentrations in MAE and MAC extracts (Table 2).

MAE extract presented the highest concentration of total phenolic compounds, followed by MAC and then UAE extract (15.9, 10.1, and 6.6 mg/g, respectively). MAE extracts presented higher concentrations of hydrolysable tannins, with granatin B being the one standing out with the highest concentration (3 mg/g). MAE extract also presented the highest concentration of total flavonoids, 6.8 mg/g, with (-)-epicatechin being the one with a higher concentration (2.4 mg/g). In opposition, the extract that presented the highest amount of phenolic acids was the MAC extract. This extract presented a total phenolic acid concentration of 4.45 mg/g, with gallic acid being the phenolic acid compound that presented the highest concentration in all three extracts (MAE—0.95 mg/g; UAE—1.19 mg/g; MAC—1.45 mg/g).

Several authors have already studied this compound and linked these molecules to several health properties of interest, such as anticancer, antidiabetic, antioxidant, and anti-inflammatory [44,45,46]. The extraction of these compounds from pomegranate leaves, which are an economically promising and abundant by-product, could be of interest for the food and pharmaceutic industry.

### 2.2. Antioxidant Activity

The present work evaluated the antioxidant activity of the three hydroethanolic leaf extracts using the cell-based assays, TBARS and CAA. The obtained results are presented in Table 3. For the TBARS assay, the results demonstrated that MAC and MAE presented the most potent TBARS inhibitory capacity, exhibiting the lowest IC_50_ values (0.83 and 0.86 μg/mL, respectively). On the contrary, UAE extract presented a IC_50_ value of 1.70 μg/mL, being the extract with lesser antioxidant potential. Nonetheless, all three extracts exhibited higher antioxidant effects than the positive control trolox (10.7, 10.9, and 5.3 times higher, for MAC, MAE, and UAE, respectively). In addition to the TBARS assay, the pomegranate leaves extracts were also submitted to intracellular ROS inhibition assay induced by dichlorodihydrofluorescein diacetate in RAW 264.7 macrophage cell lines. The results showed no ROS inhibition at the highest tested concentration (2000 µg/mL) for all the studied extracts (Table 3).

MAE and MAC extracts exhibited lower IC_50_ values and, therefore, higher antioxidant potential and are also the extracts that presented higher total phenolic compositions (15.9 and 10.1 mg/g, respectively). Our results are in agreement with the ones obtained by Derakhshan et al. [20], who studied pomegranate peel, seed, and juice. According to other authors, antioxidant activity is frequently associated with phenolic compounds concentration [47]. In our investigation, the samples with the highest content of phenolic compounds (MAE and MAC) presented lower IC_50_ values in the TBARS production assay when compared to the third extraction methodology (UAE). These data point to a probable positive association between these two measures (phenolic compound concentration and inhibition of TBARS generation). To the best of the author’s knowledge, this is the first study that investigates and analyzes the antioxidant potential of three hydroethanolic pomegranate leaf extracts obtained through two cell-based assays.

### 2.3. Cytotoxic and Hepatotoxic Activity

The cytotoxic efficacy of several pomegranate leaf extracts was investigated against four human tumor cell lines and a primary pig liver cell line (PLP2). Table 4 displays the collected qualitative properties. The results are reported as the extract concentration that inhibits cell growth by 50% (GI_50_); therefore, a lower GI_50_ value indicates more effective cytotoxic action.

All three extracts presented cytotoxic effects against all tested tumor cell lines. Gastric adenocarcinoma (AGS), followed by the colorectal tumor cells (CaCo2), showed a higher sensitivity to the three leaf extracts when compared to the remaining tumor cell lines tested (Table 4). Both AGS and CaCo_2_ cell lines presented the lowest GI_50_ values when exposed to the UAE extract (19 and 63 μg/mL, respectively). Overall, the UAE leaf extract presented a higher cytotoxic effect when compared to the other two extracts. It should be noted the existence of toxicity in relation to non-tumor liver cells (PLP2), with values of GI_50_ between 54 and 57 μg/mL (Table 4). Therefore, further studies are required to verify the cytotoxicity of pomegranate leaves against other non-tumor cells and its possible use as an effective and safe chemotherapeutic ingredient. Breast adenocarcinoma cell line (MCF-7) presented similar GI_50_ values for the three tested extracts (between 70 and 71 μg/mL). In agreement with our findings, Li et al. [25] verified that pomegranate leaf extract could inhibit H1299 lung cancer cells proliferation by inhibiting the cell cycle progression and inducing apoptosis. Most of the existent studies that evidence pomegranate as having anti-cancer effects use in general PJ or pomegranate fruit extract [48,49]. A previous in vitro study showed that the proliferation and cell growth of the MCF-7 breast cancer cell line were significantly inhibited by PJ [50]. Nonetheless, the anti-tumor potential of the fruit is not limited to the edible part. Other pomegranate plant parts have also been shown to have antiproliferative and cytotoxic properties against prostate, lung, colon, and skin cancer cell models [49]. Both Hong et al. [51] and Seidi et al. [52] verified that punicalagin and ellagic acid isolated from the pomegranate peel as well as pomegranate leaves extract significantly inhibited the proliferation of A549 and H1299 lung cancer cell lines. In particular, punicalagin, a known hydrolysable tannin present in the three studied extracts, was reported to inhibit cell viability, migration, and invasion in MCF-7 and MDA-MB-231 breast cancer cell models [53].

### 2.4. Anti-Inflammatory Activity

The Nitric oxide (NO) inhibitory effect of pomegranate leaf extracts was tested in RAW 264.7 cells, and the results are presented in Table 5. NO is a proinflammatory mediator produced by inducible nitric oxide synthase (iNOS). It is a chemical mediator involved in the inflammation process. None of the studied extracts exhibited the capacity to inhibit NO production in the murine macrophage cells (GI_50_ > 400 μg/mL).

Little has been studied about the effects of pomegranate leaf extracts when it comes to anti-inflammatory properties, but opposite to our results, De Oliveira et al. [54] verified that pomegranate leaf extract presented beneficial anti-inflammatory properties in a mouse model of asthma. In this study, they used a rat acute peritonitis model to show that pre-treatment with hydroethanolic extract prepared from pomegranate leaves derived from pomegranate leaves reduced mRNA levels of TNF-α in the LPS-induced mouse model [55]. These authors reported the presence of flavonoids such as kaempferol, luteolin, apigenin, and quercetin in the pomegranate leaf extract, which are all phenolic compounds that we also identified in our three samples. These compounds have been screened as anti-inflammatory agents due to their ability to modulate immune cells and inhibit proinflammatory cytokine production [4]. Besides NO, other inflammatory mediators such as interleukin-6, cyclooxygenase-2, prostaglandin E2, and monocyte chemoattractant protein-1 have been dose-dependently inhibited after exposure to extracts obtained from different pomegranate plant parts and individual compounds found in pomegranate leaves [56,57].

### 2.5. Skin-Beneficial Properties

**Figure 2:**
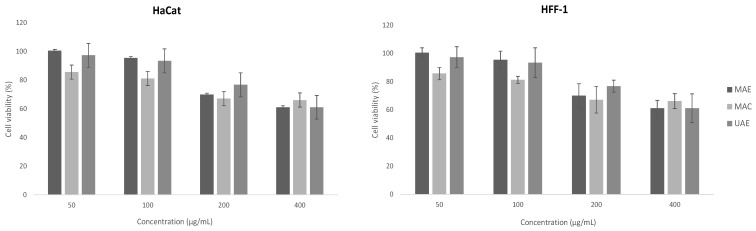
Effect on pomegranate leaves extract on viability of HFF-1 (human fibroblasts) and HaCaT (human keratinocytes) cells at different concentrations using the SRB assay. Positive control: Triton X-100 at a final concentration of 1% (*w*/*v*).

All three extracts were tested on two skin cell-lines, human fibroblasts (HFF-1) and keratinocytes (HaCaT), to assess cell viability, and the results are presented in Figure 2. As verified in those figures, in both studied cell lines, HFF-1 and HaCat, more than 50% of viability was maintained after exposure to the highest tested concentrations of each extract (400 µg/mL). An observed reduction in keratinocyte cell viability was evident in all the tested extracts compared to the fibroblast cells. In the HFF-1 cell line, 400 μg/mL for all the studied extract is the subtoxic concentration to be utilized for further studies, as more than 70% viability was maintained compared to the untreated control (media). This % viability is proposed by the International Organization for Standardization, which states that <70% cell viability compared to the untreated control is considered a non-cytotoxic effect. On the other hand, the subtoxic concentration for the MAE and UAE extracts on HaCaT cells is 200 μg/mL, where more than 70% cell viability was maintained.

As shown in Table 5, the MAE extract revealed the highest tyrosinase inhibition potential, closely followed by the MAC extract. Specifically, both extracts, at the highest tested concentration (300 µg/mL), provided over 30% tyrosinase inhibitory activity. The UAE extract presented the least tyrosinase inhibitory potential.

Overall, the results showed that although viability differs slightly in the two tested cell lines, the effect showed promising safety potential, permitting its potential use as an ingredient in dermatological formulation development. The tyrosinase enzyme is the rate-limiting enzyme that catalyzes two major steps involved in melanogenesis. The overproduction of melanin is associated with hyperpigmentation and other skin disorders, hence the constant search for naturally derived biomolecules that inhibit tyrosinase activity. In the present work, the tyrosinase inhibitory effect of the three studied extracts was assessed using L-DOPA as a substrate. Ellagic acid and punicalagin are two pomegranate bioactive components that enhance skin health by inhibiting tyrosinase and beginning anti-inflammatory and anti-fungal activities [58,59,60], but to the best of the author’s knowledge, those components from pomegranate leaf extracts have not been tested for this purpose. Nonetheless, Yoshimura et al. [60] discovered that pomegranate peel extract containing 90% ellagic acid could reduce tyrosinase activity, inhibit melanocyte proliferation and synthesis, and thus lower the risk of skin cancer. Ellagic acid from pomegranate peel has also been studied for its ability to stimulate dermal fibroblast proliferation and collagen synthesis while inhibiting the activity of the skin’s main collagen-degrading enzymes [49].

### 2.6. Antimicrobial Activity

In this study, the antibacterial and antifungal properties of the different leaf extracts were accessed. The minimum inhibitory concentration (MIC), minimum bactericidal concentration (MBC), and minimum fungicidal concentration (MFC) values are shown in Table 6 and Table 7. All the studied extracts had the capacity to inhibit bacterial growth of the clinical strains screened (0.03125 to 10 mg/mL). The MIC values obtained for both Gram-positive and Gram-negative bacteria were similar. *K. pneumoniae* presented the lowest MIC values (0.6 mg/mL), and as such, revealed the highest susceptibility to the three tested pomegranate leaves’ extracts. *Morganella morganii*, an opportunistic pathogen that colonizes post-operative wounds and causes urinary tract infections, was also effectively inhibited by MAE extract. Although the antibacterial potential is influenced by the bacteria strain under analysis, the MAE and MAC extracts were the ones with the most promising activity, exhibiting the lowest MIC values for the five Gram-negative strains tested. Regarding the Gram-positive strains, all extracts showed some antibacterial inhibition activity. MRSA was revealed to be the most sensitive bacteria to our different leaf extracts, where the MIC values vary between 0.3 and 0.6 mg/mL. In accordance with other authors, these results could be linked to the fact that phenolic compounds inhibit bacterial proliferation and swimming ability, damaging the integrity and stability of the bacterial cell membrane [61]. Pisoschi et al. [62] has explained that polyphenols interact with membrane proteins, disrupting cell membrane functions, and having an impact on nutrient absorption, the electron transfer system, enzyme activity, and protein and nucleic acid synthesis. When the cell membrane’s integrity is compromised, polyphenols, such as punicalagin, enter the cytoplasm and bind to the target gene domain, causing regulatory networks to become disorganized and eventually causing bacterial damage.

As for the food contaminants (Table 7), as with the clinical strains, the three extracts showed the ability to inhibit bacterial growth and did not cause the bacterial strains’ death. Regarding Gram-negative bacteria, we verified that *Salmonella enterica* was the one presenting higher sensibility to the extracts, with the MAE being most effective in inhibiting its growth. *Staphyloccocus aureus* was the Gram-positive strain that showed the most significant inhibitory response, when compared with the other two strains. In this case, as with the clinical strains, MAE was the extract showing the best inhibition results.

To the best of our knowledge, no studies have been performed on pomegranate leaf extract and food contaminants’ inhibition growth, but several studies do exist about pomegranate peel extract and the use of pomegranate juice to inhibit bacterial growth. PP is the most studied by-product, and according to Aguilera-Carbo et al. [63] and Akhtar et al. [64], pomegranate peel seems to exert antibacterial effects on a wide number of foodborne pathogens and infectious microorganisms (e.g., *Staphylococcus aureus*, *Escherichia coli*, *Klebsiella pneumoniae*, *Bacillus subtilis*). Tayel et al. [65] and Kharchoufi et al. [66] explained that the antibacterial activity of pomegranate peel extract could be attributed to the combined effect of its constituents, which include powerful compounds with antibacterial, antifungal, and antioxidant properties, such as kaempferol, castalagin, granatin, gallocatechin, quercetin, and other phytochemical compounds present in minor quantities. Those compounds were also identified in our leaves’ extracts, suggesting a similar antimicrobial potential. Pomegranate peel, as well as the studied hydroethanolic extracts, contain a punicalagin compound, which is said to have powerful antibacterial properties against *Pseudomonas aeruginosa* and *Staphyloccocus aureus.* According to Nuamsetti et al. [67], not only does the peel seem to present those properties, pomegranate arils, including seeds, also present antibacterial properties against *Bacillus subtilis*, *Staphylococcus aureus*, *Salmonella typhimurium*, and *Escherichia coli*. Okan et al. [68] also investigated the antifungal properties of pomegranate seed oil (PSO) against five different plant pathogens and discovered that at a concentration of 1000 ppm, PSO inhibited only about 20% mycelial growth.

The antifungal activity was also evaluated (Table 6), and *A. brasiliensis* appeared to be the most sensitive to the tested extracts, with MIC values ranging from 0.6 to 1.25 mg/mL. The MAE extract presented the lowest MIC values and, therefore, has the best antifungal potential, even though the other two tested extracts presented a relative inhibition potential. In the same line of fungal study, Bhinge et al. [69] studied the alcoholic leaf extract of pomegranate and found that it inhibited the growth of major fungal pathogens that cause dandruff, such as *Candida albicans*, *Aspergillus niger*, and *Penicillium notatum*. As a possible explanation, and according to some authors, the induction of host defense mechanisms and changes in fungal cell membrane permeability could be attributed to the polyphenol content [70].

Overall, pomegranate leaf extracts constitute a promising natural source of antimicrobial compounds. This knowledge testifies to their capacity to inhibit several multi-resistant bacteria and fungi strains responsible for causing several public health concerns. The obtained results encourage further study, enhancing its bioactive potential and so supporting its sustainable exploitation.

## 3. Materials and Methods

### 3.1. Plant Material and Reagents

The pomegranate leaves were collected from Quinta do Prado, Vale Frechoso, Vila Flor. The leaves collected were the ones discarded by the company after harvesting. The company’s name is Acushla S.A. and the varieties they produce are Bigfull and Wonderfull. The samples were freeze-dried and reduced to a fine powder (~20 mesh) using a domestic electric blender. They were then homogenized to obtain representative samples.

### 3.2. Extraction Procedures

Three extraction methodologies were performed: maceration (MAC), ultrasound-assisted extraction (UAE), and microwave-assisted extraction (MAE). In all referred extractions, a hydroethanolic solvent was used (EtOH/H_2_O, 60:40, *v*/*v*) and a solid–liquid ratio of 1:20 was maintained.

MAC was performed using 2 g of *P. granatum* L. leaves with 40 mL of solvent at room temperature. The sample was kept under continuous electromagnetic stirring, and after 1 h, the extract solution was recovered by filtration.

UAE was carried out using an ultrasonic system (Ultrasonic homogenizer, model CY-500, Optic Ivymen System, Barcelona, Spain) equipped with a titanium probe. For this extraction technique, 5 g of dried pomegranate leaves sample was extracted in 100 mL of solvent for 5 min at 350 W power.

The MAE process was performed in a microwave Digestion system (Speedwave Xpert, Berghof, Eningen, Germany); 1 g of the sample was extracted with 20 mL of solvent during 15 min at 80 °C, with a 350 W power and a ramp time of 7 min.

The organic solutions were filtered through Whatman No.4 filter paper and concentrated under low pressure at 40 °C (rotary evaporator Buchi R-210, Flawil, Switzerland). The aqueous phase was frozen and lyophilized (FreeZone 4.5, Labconco, Kansas City, MO, USA).

### 3.3. Chromatographic Analysis of Chemical Constituents

The obtained extracts were redissolved in EtOH/H_2_O (60:40, *v*/*v*), at a final concentration of 10 mg/mL, and filtered using 0.22 µm nylon syringe filters. High-performance liquid chromatography combined with diode array detection and electrospray ionization mass spectrometry (HPLC-DAD-ESI/MS^n^) was used to identify the profile of phenolic compounds according to the chromatographic conditions previously described by Bessada et al. [71]. Data acquisition, processing, and interpretation were performed with Xcalibur software version 2.2 (Thermo Finnigan, San Jose, CA, USA). The tentative identification of phenolic compounds was inferred by comparing the retention periods (Rt), wavelength of maximum absorption (λ_max_), pseudomolecular ion ([M–H]^−^), UV-Vis spectra, mass spectra, and patterns of the ion breakdown (MS^2^) to those of commercial standards and the information found in the literature. Quantification was performed by measuring the peak area and utilizing calibration curves created for each commercially available standard (Extrasynthèse, Genay, France). The results were expressed in mg per g of extract.

### 3.4. Bioactive Properties

#### 3.4.1. Antioxidant

The antioxidant activity of the extracts was assessed using two cell-based assays: the thiobarbituric acid reactive substance production inhibition (TBARS) and cellular antioxidant assay (CAA).

TBARS assay: This cell-based assay was followed according to the previous report by Mandim et al. [72]. The *P. granatum* leaf extracts were re-dissolved in water to obtain the final concentrations to be tested (between 0.0003051 and 0.1953 μg/mL). Trolox was used as a positive control, and the results were presented as the concentration of extract that inhibited the oxidative process by 50% (IC_50_, µg/mL).

Cellular antioxidant activity (CAA): Pomegranate leaves’ extracts were re-dissolved in water at 8 mg/mL and submitted to successive dilutions with 2′,7′-dichlorohydrofluorescein (DCFH) prepared with ethanol and diluted with HBSS (50 μM), acquired from Hyclone company (Logan, Utah, USA), to obtain the final concentrations to be tested (32.5–2000 μg/mL). This cell-based process was carried out as stated by Pinela et al. [73] using a murine macrophage cell line (RAW 246.7) acquired from Leibniz-Institute DSMZ. The cells were maintained with DMEM supplemented with 10% heat-inactivated FBS, glutamine (2 mM), penicillin (100 U/mL), and streptomycin (100 μg/mL) and left to proliferate in an incubator at 37 °C, 5% CO_2_, and humified atmosphere (Heal Force CO_2_ Incubator, Shanghai Lishen Scientific Equipment Co, Ltd., Shangai, China). The cells were used only when 70 to 80% of confluence was achieved. Cells were seeded at 7 × 10^4^ cells/mL in 96 black well plates (SPL Life Sciences, Korea). After 48 h of incubation, cells were treated with different extract concentrations and incubated for 1 h according to the conditions described above. The plates were then washed with a saline solution (HBSS, 100 µL) and a solution of 2,2′Azobis (2 methylpropionamide) (AAPH) (100 μL; 600 μM) was added. Fluorescence was read every 5 min for 1 h (Biotek FLX800, BioTek Instruments, Inc., Winooski, VT, USA) at 485 nm excitation and 538 nm emission. The obtained results were expressed as an inhibition percentage of the oxidative reaction at the maximum concentration tested. Quercetin was used as the positive control, and DCFH and DMEM culture medium were used as the negative control.

#### 3.4.2. Cytotoxicity and Hepatotoxicity

The cytotoxic activity of the extract solutions (0.3906–400 μg/mL in water) was determined using the sulforhodamine B colorimetric test (bought from Sigma-Aldrich, Saint Louis, MO, USA), as previously described by Barros et al. [74]. Four human cell-lines were tested: breast adenocarcinoma (MCF-7), non-small cell lung carcinoma (NCI-H460), gastric adenocarcinoma (AGS), and colorectal adenocarcinoma (CaCo-2), all purchased from Leibniz-Institut DMSZ—Deutsche Sammlung von Mikroorganiismen und Zellkulturen GmbH, Braunschweig, Germany. A non-tumor cell line was also tested, a primary culture PLP2, previously established in the lab using pig liver, according to the methodology described by Mandim et al. [75]. The cell lines assessed were routinely maintained as adherent cell cultures in RPMI-1640 media supplemented as previously described in Section 3.4.1. All extract concentrations (10 μL) were incubated for 72 h with the cell-lines suspension, at 1 × 10^4^ cells per well in 96well plates. The positive control used was ellipticine, and the results were expressed as the extract concentration that inhibited 50% of the cell proliferation (GI_50_ values, μg/mL).

#### 3.4.3. Anti-Inflammatory Activity

The extracts’ anti-inflammatory activity was determined by measuring the ability of all three extracts to inhibit the production of nitrite oxide (NO). As reported by Mandim et al. [76], a murine macrophage cell line (RAW 264.7) was used and stimulated with lipopolysaccharide (LPS) to induce a response (Sigma-Aldrich, Saint Louis, MO, USA). The extracts of pomegranate leaves were redissolved in water to achieve an 8 mg/mL solution. This solution was then successively diluted to obtain different concentrations (between 6.25 and 400 g/mL). The positive control was commercially purchased: dexamethasone from Sigma-Aldrich, Saint Louis, MO, EUA. Cells with and without LPS were used as a negative control. The results were expressed as the extract concentration that inhibited 50% of NO production (IC_50_, μg/mL).

#### 3.4.4. Skin-Beneficial Properties

Cell viability assay in skin cell lines: The SRB assay was used to assess cell viability in human fibroblasts (HFF-1, purchased from Cell Line Service, Germany) and keratinocytes (HaCaT, obtained from ATCC) following the previously described protocol with slight modifications [77]. Cells were seeded in 96-well plates at a density of 2 × 10^4^ cells/well and incubated for 24 h. Different concentrations of extracts were added (37.5–300 µg/mL) to each well. After the incubation period, an ice-cold solution of trichloroacetic acid (TCA) (10%, *w*/*v*) was added to each well followed by incubation for 1 h at 4 °C. Afterward, the microplates were washed with water and dried at room temperature. A solution of SRB (0.057%, *w*/*v*) was then added to each well and left to dry at room temperature for 30 min. Wells were washed three times with an acetic acid solution (1%, *v*/*v*) and left to dry at room temperature. Finally, the SRB was solubilized with Tris (10 mM, 200 μL) and the absorbance was measured at 540 nm (SPECTROstar Nano Multi Detection Micro Plate Reader; BMG Labtech, Ortenberg, Germany). The positive control used was Triton X-100 at a final concentration of 1% (*w*/*v*). The results were expressed in viability percentage. According to the International Organization for Standardization, a cell viability higher than 70% is considered as to have no cytotoxic effect.

Tyrosinase inhibitory activity: The tyrosinase enzyme inhibition activity was evaluated using L-DOPA (5 mM) as the substrate and MBTH (20.7 mM) (both acquired from Sigma-Aldrich, Saint Louis, MO, EUA) as the chromogenic stabilizing agent in 96-well microplates according to the procedure previously described by Winder et al. [78]. Briefly, different concentrations of the tested samples (10 µL; between 37.5 and 300 μg/mL), phosphate buffer (pH 7.1, 0.1 M), L-DOPA (60 µL), and MBTH (87 µL) were mixed and pre-incubated at 25 °C for 10 min. Subsequently, 6 µL of mushroom tyrosinase enzyme (142 units/mL), purchased from Sigma-Aldrich, Saint Louis, MO, EUA, was added to each well and the plates were incubated for 30 min at 25 °C. The formation of the dopaquinone-MBTH complex was evaluated at 505 nm, using a microplate spectrophotometer (SPECTROstar Nano Multi-Detection Microplate Reader; BMG Labtech, Ortenberg, Germany). 4-butylresorcinol was used as positive control (bought from Sigma-Aldrich, Saint Louis, MO, USA). The results were presented as the percentage of tyrosinase enzyme inhibition and were calculated using the following equation:

$$\%\;tyrosinase\;inhibition=\left(\frac{Absorbance\;Control-Absorbance\;Sample}{Absorbance\;Control}\right)*\;100$$

#### 3.4.5. Antimicrobial Activity

The antibacterial activity of extracts was tested against several clinical strains and foodborne pathogens. The clinical bacteria were obtained from the Local Health Unit of Bragança and the Hospital Center of Trás-os-Montes and Alto- Douro, Vila Real, Northeast of Portugal. Food contaminants and antifungal strains were purchased from Frilabo, Porto, Portugal. The foodborne pathogens and clinical strains used were Gram-negative and -positive bacteria, as mentioned in Table 6 and Table 7. The antifungal potential was evaluated using two fungal strains also presented in Table 6 and Table 7. The clinical and food contaminants assays were performed by the microdilution method in a 96-well microplate, previously described by Pires et al. [79]. To maintain exponential growth, the foodborne pathogens were incubated into a new medium at 37 °C for 24 h before using them. Micromycetes were grown on malt agar, stored at 4 °C before being transferred to a new medium and incubated at 25 °C for 72 h [80]. The extracts’ minimum inhibitory concentrations (MICs), and minimal bactericidal (MBC) and fungicidal concentrations (MFC) were determined. The methodology followed was reported by Pires et al. [79]. Briefly, MIC determination was achieved using a colorimetric method and was determined as the lowest extract concentration that avoided the medium color change (from yellow to pink). The MBC and MFC were established as the lowest extracts concentration necessary to kill bacteria and fungi strains, respectively.

### 3.5. Statistical Analysis

Differences among samples were analyzed using SPSS Statistics software (IBM SPSS Statistics for Mac OS, Version 26.0. Armonk, NY, USA: IBM Corp.). The results were subject to an analysis of variance (ANOVA), while Tukey’s HSD test (α = 0.05) was used to assess the significant differences between the samples. For the comparison between the two samples, a two-tailed paired Student’s *t*-test was applied to assess the statistical differences at a 5% significance level.

## 4. Conclusions

Pomegranate leaves are considered biowaste, even though they contain structurally diverse bioactive molecules. These compounds have been widely studied due to their observed biological effects. In the present work, the highest content of phenolic compounds was obtained in the MAE extract. Gallic acid, epicatechin, and granatin B were the most abundant compounds detected in all three extraction methodologies studied. Better antioxidant activity for TBARS was obtained for MAE and MAC extracts. All the studied extracts showed no discernible negative effects on the studied skin cell lines and presented cytotoxicity against all tumor cell lines tested. However, in this latter case, the UAE extract had the lowest GI_50_ values. Through this research, we also verified that MAE pomegranate leaf extract was the one that presented the best bacteriostatic effect against clinical and food pathogens. This study improves knowledge of pomegranate waste disposal methods and enhances their potential utilization in skin health and food science research. Overall, these findings provide experimental evidence supporting the potential use of pomegranate leaves as a functional bioactive ingredient. They could also be used as a rich source of molecules of interest in diverse areas of industry. Based on these findings, further studies on the molecular mechanism underlying the demonstrated bioactive properties, and the most important contributing compounds, should be further studied.

## Figures and Tables

**Table 1:** Retention time (Rt), the wavelength of maximum absorption (λ_max_), mass spectral data, and tentative identification of the phenolic compounds present in the three leaf extracts.

Peak	R*t* (min)	λ_max_ (nm)	[M–H]^−^ (*m*/*z*)	MS^2^ (*m*/*z*)	Tentative Identification
1	3.84	285	169	125(100)	Gallic acid
2	4.19	322	353	191(100), 179(69), 161(7), 135(51)	3-*O*-Caffeoylquinic acid
3	4.37	320	345	183(100), 169(33)	Methyl gallate-hexoside
4	4.71	320	341	179(100)	Caffeic acid hexoside
5	5.08	283	355	209(100), 191(62)	Glucaric acid rhamnoside
6	5.21	333	353	191(100), 179(4), 161(5), 135(3)	5*-O*-Caffeoylquinic acid
7	5.36	280	289	245(100)	(+)-Catechin
8	5.58	282	355	209(100), 191(62)	Glucaric acid deoxyhexoside
9	5.90	361	477	301(100)	Methyl ellagic acid hexoside
10	6.41	325	401	269(100)	Apigenin-*O*-pentoside
11	6.65	282	289	245(100)	(-)-Epicatechin
12	7.86	319	355	193(100)	Ferulic acid hexoside
13	8.61	358	609	301(100)	Ellagic acid-(*p*-coumaroyl)hexoside
14	9.53	349	433	301(100)	Ellagic acid pentoside
15	11.62	345	447	301(100)	Methyl ellagic acid pentoside
16	12.34	359	491	315(100)	Isorhamnetin-*O*-glucuronide
17	12.74	361	609	301(100)	Quercetin-3-*O*-rutinoside
18	13.14	335	593	285(100)	Luteolin-7-*O*-rutinoside
19	13.51	357	463	301(100)	Quercetin-3-*O*-glucoside
20	14.01	352	477	301(100)	Quercetin-*O*-glucuronide
21	14.17	334	447	285(100)	Luteolin-7-*O*-glucoside
22	14.61	335	461	285(100)	Luteolin-*O*-glucuronide
23	15.87	354	505	301(100)	Quercetin-*O*-acetyl-glucoside
24	16.41	352	447	315(100)	Isorhamnetin-*O*-pentoside
25	17.05	344	447	285(100)	Kaempferol-3-*O*-glucoside
26	17.31	349	461	315(100)	Isorhamnetin-*O*-rhamnoside
27	18.16	331	445	269(100)	Apigenin-*O*-glucuronide
28	18.82	332	951	933(100), 631(12), 613(9), 301(48)	Granatin B
29	19.28	339	1083	1065(51), 1021(38), 807(11), 721(100), 575(2)	Punicalagin
30	20.98	338	799	767(33), 461(100), 301(21)	Lagerstannin A
31	21.78	335	785	633(100), 615(45), 483(33), 301(12)	Digalloyl-HHDP-hexoside
32	23.95	288	783	765(100), 301(61), 275(12)	Pedunculagin (bis-HHDP-hex)
33	27.02	333	935	783(100), 301(23)	Casuarictin

**Table 2:** Quantification (mg/g) of the phenolic compounds in the pomegranate leaf extracts.

Peak	Tentative Identification	MAE	UAE	MAC
1	Gallic acid	0.95 ± 0.01 ^c^	1.19 ± 0.01 ^b^	1.45 ± 0.01 ^a^
2	3-*O*-Caffeoylquinic acid	0.31 ± 0.01 ^a^	0.101 ± 0.001 ^c^	0.160 ± 0.001 ^b^
3	Methyl gallate-hexoside	0.151 ± 0.001 ^c^	0.229 ± 0.002 ^a^	0.212 ± 0.001 ^b^
4	Caffeic acid hexoside	0.0560 ± 0.0002 ^c^	0.16 ± 0.01 ^b^	0.17 ± 0.01 ^a^
5	Glucaric acid rhamnoside	0.77 ± 0.03	n.d.	n.d.
6	5-*O*-Caffeoylquinic acid	0.012 ± 0.001 ^c^	0.67 ± 0.01 ^b^	0.91 ± 0.01 ^a^
7	(+)-Catechin	0.92 ± 0.03	n.d.	n.d.
8	Glucaric acid deoxyhexoside	0.61 ± 0.02	n.d.	n.d.
9	Methyl ellagic acid hexoside	0.060 ± 0.003 ^c^	0.161 ± 0.003 ^b^	0.434 ± 0.003 ^a^
10	Apigenin-*O*-pentoside	0.189 ± 0.002 ^b^	0.119 ± 0.002 ^c^	0.266 ± 0.001 ^a^
11	(-)-Epicatechin	2.40 ± 0.03 ^a^	0.70 ± 0.02 ^c^	0.83 ± 0.02 ^b^
12	Ferulic acid hexoside	0.043 ± 0.002	tr.	tr.
13	Ellagic acid-(*p*-coumaroyl)hexoside	0.130 ± 0.003 *	n.d.	0.519 ± 0.002 *
14	Ellagic acid pentoside	0.273 ± 0.003 ^a^	0.0060 ± 0.0003 ^c^	0.202 ± 0.002 ^b^
15	Methyl ellagic acid pentoside	0.219 ± 0.001 ^a^	0.116 ± 0.005 ^a^	0.391 ± 0.002 ^a^
16	Isorhamnetin-*O*-glucuronide	0.120 ± 0.002 ^a^	0.047 ± 0.001 ^a^	0.48 ± 0.01 ^a^
17	Quercetin-3-*O*-rutinoside	0.57 ± 0.02 ^a^	0.227 ± 0.002 ^a^	0.316 ± 0.002 ^a^
18	Luteolin-7-*O*-rutinoside	0.68 ± 0.01	n.d.	n.d.
19	Quercetin-3-*O*-glucoside	0.199 ± 0.001 ^a^	0.092 ± 0.002 ^b^	0.199 ± 0.001 ^a^
20	Quercetin-*O*-glucuronide	0.182 ± 0.002 ^c^	1.20 ± 0.02 ^a^	0.48 ± 0.02 ^b^
21	Luteolin-7-*O*-glucoside	0.22 ± 0.01	n.d.	n.d.
22	Luteolin-*O*-glucuronide	0.100 ± 0.002 ^a^	0.1 ± 0.1 ^b^	0.174 ± 0.001 ^c^
23	Quercetin-*O*-acetyl-glucoside	0.207 ± 0.001 ^b^	0.140 ± 0.002 ^c^	0.258 ± 0.001 ^a^
24	Isorhamnetin-*O*-pentoside	0.56 ± 0.02 ^a^	0.039 ± 0.001 ^c^	0.191 ± 0.001 ^b^
25	Kaempferol-3-*O*-glucoside	0.067 ± 0.002 ^c^	0.58 ± 0.01 ^b^	0.61 ± 0.02 ^a^
26	Isorhamnetin-*O*-rhamnoside	0.097 ± 0.003 ^c^	0.1040 ± 0.0004 ^b^	0.250 ± 0.001 ^a^
27	Apigenin-*O*-glucuronide	0.269 ± 0.001 ^b^	0.217 ± 0.001 ^c^	0.52 ± 0.01 ^a^
28	Granatin B	3.0 ± 0.1 ^a^	0.133 ± 0.001 ^b^	0.26 ± 0.01 ^c^
29	Punicalagin	0.088 ± 0.001 ^b^	0.057 ± 0.002 ^c^	0.119 ± 0.001 ^a^
30	Lagerstannin A	0.185 ± 0.001 ^a^	0.063 ± 0.001 ^c^	0.081 ± 0.001 ^b^
31	Digalloyl-HHDP-hexoside	0.089 ± 0.002 *	n.d.	0.177 ± 0.001 *
32	Pedunculagin (bis-HHDP-hex)	0.47 ± 0.01 ^a^	0.054 ± 0.002 ^c^	0.224 ± 0.001 ^b^
33	Casuarictin	1.73 ± 0.01 ^a^	0.086 ± 0.001 ^b^	0.21 ± 0.02 ^c^
	Total phenolic acids	3.7 ± 0.1 ^b^	2.52 ± 0.03 ^c^	4.45 ± 0.04 ^a^
	Total flavonoids	6.8 ± 0.1 ^a^	3.6 ±0.1 ^c^	4.6 ± 0.1 ^b^
	Total hydrolyzable tannins	5.48 ± 0.02 ^a^	0.394 ± 0.004 ^c^	1.08 ± 0.01 ^b^
	Total phenolic compounds	15.9 ± 0.3 ^a^	6.6 ± 0.1 ^c^	10.1 ± 0.1 ^b^

n.d.—not detected; tr.—traces. Results are presented as mean ± standard deviation. Different letters in the same line correspond to significant differences according to Tukey’s honest significance (HSD) test (*p* < 0.05). * Mean statistical differences obtained by Student’s t-test, *p*-value < 0.01. MAE—microwave-assisted extraction; UAE—ultrasound-assisted extraction; MAC—maceration. Calibration Curves used: Peaks 1, 3, 28–33: gallic acid (y = 131,538x + 292,163; R^2^ = 0.9969; LOD = 8.05 μg/mL; LOQ = 24.41 μg/mL); Peaks 2, 6: chlorogenic acid (y = 168,823x − 161,172; R^2^ = 0.9999; LOD = 0.20 µg/mL; LOQ = 0.68 µg/mL); Peaks 9, 13–15: ellagic acid (y = 26,719x − 317,255; R^2^ = 0.9986; LOD = 0.41 µg/mL; LOQ = 1.22 µg/mL); Peaks 10 and 27: apigenin 7-O-glucoside (y = 10,683x − 45,794; R^2^ = 0.999; LOD = 0.10 μg/mL; LOQ = 0.53 μg/mL); Peaks 7 and 11: catechin (y = 84,950x − 23,200; R^2^ = 0.9999; LOD 0.17 μg/mL; LOQ 0.68 μg/mL); Peak 12: ferulic acid (y = 63,326x − 185,462; R^2^ = 0.999; LOD = 0.20 μg/mL; 1.01 μg/mL); Peak 4: (y = 90,492x − 29,265; R^2^ = 0.9986; LOD = 0.41 µg/mL; LOQ = 1.22 µg/mL); Peaks 5 and 8: hydroxybenzoic acid (y = 20,800x + 41,309; R^2^ = 0.9986; LOD = 0.41 µg/mL; LOQ = 1.22 µg/mL); Peaks 16, 19, 20, 23–26: quercetin 3-O-glucoside (y = 34,843x − 160,173; R^2^ = 0.9998; LOD = 0.21 µg/mL; LOQ = 0.71 µg/mL); Peak 17: quercetin-3-O-rutinoside (y = 13,343x + 7675; R^2^ = 0.9998; LOD = 0.1 μg/mL; LOQ = 0.65 μg/mL); Peaks 18, 21, 22: luteolin-7-O-glucoside (y = 43,453x − 1354.5; R^2^ = 0.998; LOD = 0.40 µg/mL; LOQ = 0.88 µg/mL). Total phenolic acids—sum of the amount of compounds 1–6, 8, 9, 12–15; total flavonoids—sum of 7, 10, 11, 16–27; total hydrolysable tannins—sum of the amount 28–33; total phenolic compounds—sum of the amounts of all thirty-three compounds.

**Table 3:** Antioxidant properties of *P. granatum* leaf hydroethanolic extracts.

Antioxidant Activity				
	MAE	UAE	MAC	Positive Control
TBARS (IC_50_ values, μg/mL)	0.856 ± 0.005 ^b^	1.70 ± 0.02 ^a^	0.83 ± 0.01 ^c^	9.1 ± 0.3
CAA (% inhibition at 2 mg/mL)	>2000	>2000	>2000	95 ± 5

Results are presented as mean ± standard deviation. Different letters in the same column correspond to significant differences (*p* < 0.05). MAE—microwave-assisted extraction; UAE—ultrasound-assisted extraction; MAC—maceration. IC_50_ values correspond to the extract concentration that inhibits 50% of the oxidation and inflammatory process. Trolox: TBARS. Quercetin (% inhibition at 0.3 μg/mL): CAA.

**Table 4:** Cytotoxic and hepatotoxic properties of *P. granatum* leaf hydroethanolic extracts.

Cytotoxic Activity (GI_50_ Values, μg/mL)				
	MAE	UAE	MAC	Ellipticine
MCF-7 (breast adenocarcinoma)	71 ± 6 ^a^	71 ± 7 ^a^	70 ± 6 ^a^	1.02 ± 0.2
NCI–H460 (non-small lung carcinoma)	76 ± 1 ^a^	66 ± 5 ^b^	67 ± 4 ^b^	1.01 ± 0.1
AGS (gastric adenocarcinoma)	26.3 ± 0.2 ^a^	19 ± 1 ^c^	21 ± 1 ^b^	1.23 ± 0.03
CaCo-2 (colorectal adenocarcinoma)	66.7 ± 0.3 ^a^	60 ± 3 ^b^	61 ± 2 ^b^	1.21 ± 0.02
PLP2 (porcine liver primary cells)	57 ± 4 ^a^	54 ± 4 ^a^	54 ± 4 ^a^	1.4 ± 0.1

Results are presented as mean ± standard deviation. Different letters in the same column correspond to significant differences (*p* < 0.05). MAE—microwave-assisted extraction; UAE—ultrasound-assisted extraction; MAC—maceration. GI_50_ values correspond to the concentration that causes 50% inhibition of cell proliferation. AGS—human gastric adenocarcinoma; MCF-7—human breast adenocarcinoma; NCI-H460—human lung carcinoma; CaCo-2—colorectal adenocarcinoma; PLP2—primary culture of non-tumoral pig liver cells.

**Table 5:** Anti-inflammatory and anti-tyrosinase properties of *P. granatum* leaf hydroethanolic extracts.

	MAE	UAE	MAC	Positive Control
Anti-inflammatory activity (IC_50_ values, μg/mL)	>400	>400	>400	6.3 ± 0.4
Anti-tyrosinase activity (% inhibition at 300 μg/mL)	33.1 ± 1.5 ^a^	12 ± 2 ^b^	30 ± 4 ^a^	20 ± 0.74

Results are presented as mean ± standard deviation. Different letters in the same column correspond to significant differences (*p* < 0.05). MAE—microwave-assisted extraction; UAE—ultrasound-assisted extraction; MAC—maceration. Positive controls—Dexamethasone: anti-inflammatory activity; 4-butylresorcinol: anti-tyrosinase activity.

**Table 6:** Antibacterial and antifungal activity of *P. granatum* leaf hydroethanolic extracts against clinical strains.

	MAE		UAE		MAC		Ampicillin		Imipenem		Vancomycin		Ketoconazole	
Antibacterial activity	MIC	MBC	MIC	MBC	MIC	MBC	MIC	MBC	MIC	MBC	MIC	MBC	MIC	MFC
*Gram-negative bacteria*														
* Escherichia coli*	2.5	>10	1.25	>10	1.25	>10	<0.15	<0.15	<0.0078	<0.0078	n.t.	n.t.	n.t.	n.t.
* Klebsiella* * pneumoniae*	0.6	>10	0.6	>10	0.6	>10	10	>10	<0.0078	<0.0078	n.t.	n.t.	n.t.	n.t.
* Morganella morganii*	0.6	>10	2.5	>10	2.5	>10	>10	>10	<0.0078	<0.0078	n.t.	n.t.	n.t.	n.t.
* Proteus mirabilis*	1.25	>10	1.25	>10	0.6	>10	<015	<0.15	<0.0078	<0.0078	n.t.	n.t.	n.t.	n.t.
* Pseudomonas aeruginosa*	2.5	>10	2.5	>10	2.5	>10	>10	>10	0.5	1	n.t.	n.t.	n.t.	n.t.
*Gram-positive bacteria*														
* Enterococcus faecalis*	10	>10	10	>10	10	>10	<0.15	<0.15	n.t.	n.t.	<0.0078	<0.0078	n.t.	n.t.
* Listeria monocytogenes*	2.5	>10	5	>10	2.5	>10	<0.15	<0.15	<0.0078	<0.0078	n.t.	n.t.	n.t.	n.t.
MRSA	0.3	>10	0.6	>10	0.6	>10	<0.15	<0.15	n.t.	n.t.	0.25	0.5	n.t.	n.t.
Antifungal activity	MIC	MFC	MIC	MFC	MIC	MFC	MIC	MBC	MIC	MBC	MIC	MBC	MIC	MFC
* Aspergillus brasiliensis (ATCC 16404)*	0.6	>10	1.25	>10	2.5	>10	n.t.	n.t.	n.t.	n.t.	n.t.	n.t.	0.06	0.125
* Aspergillus fumigatus (ATCC 204305)*	10	>10	10	>10	>10	>10	n.t.	n.t.	n.t.	n.t.	n.t.	n.t.	0.5	1

n.t.—not tested; MAE—microwave-assisted extraction; UAE—ultrasound-assisted extraction; MAC—maceration; MIC—minimal inhibitory concentration; MBC—minimal bactericidal concentration; MFC—minimal fungicidal concentration. Antibacterial positive controls tested—Ampicillin at 10 mg/mL, Imipenem at 1 mg/mL, and Vancomycin—1 mg/mL. Antifungal positive control tested—Ketoconazole at 1 mg/mL. MRSA—Methicillin-resistant *Staphylococcus aureus.* All extracts were tested at a maximum concentration of 10 mg/mL.

**Table 7:** Antibacterial activity of *P. granutum* leaf hydroethanolic extracts against food contaminants.

	MAE		UAE		MAC		Streptomycin		Methicillin		Ampicillin	
Antibacterial activity	MIC	MBC	MIC	MBC	MIC	MBC	MIC	MBC	MIC	MBC	MIC	MBC
*Gram-negative bacteria*												
* Enterobacter cloacoa (ATCC 49741)*	2.5	>10	1.25	>10	1.25	>10	0.007	0.007	n.t.	n.t	0.15	0.15
* Escherichia coli (ATCC 25922)*	1.25	>10	5	>10	2.5	>10	0.01	0.01	n.t.	n.t.	0.15	0.15
* Pseudomonas aeruginosa (ATCC 9027)*	2.5	>10	2.5	>10	2.5	>10	0.06	0.06	n.t.	n.t.	0.63	0.63
* Salmonella enterica (ATCC 13076)*	0.6	>10	1.25	>10	1.25	>10	0.007	0.007	n.t.	n.t.	0.15	0.15
* Yersinia enterocolitica (ATCC 8610)*	5	>10	5	>10	10	>10	0.007	0.007	n.t.	n.t.	0.15	0.15
*Gram-positive bacteria*												
* Bacillus cereus (ATCC 11778)*	10	>10	10	>10	10	>10	0.007	0.007	n.t.	n.t.	n.t.	n.t.
* Listeria monocytogenes (ATCC 19111)*	2.5	>10	2.5	>10	2.5	>10	0.007	0.007	n.t.	n.t.	0.15	0.15
* Staphylococcus aureus (ATCC 25923)*	0.3	>10	0.6	>10	0.6	>10	0.007	0.007	0.007	0.007	0.15	0.15

n.t.—not tested; MAE—microwave-assisted extraction; UAE—ultrasound-assisted extraction; MAC—maceration; MIC—minimal inhibitory concentration; MBC—minimal bactericidal concentration. Antibacterial positive controls tested—Streptomycin at 1 mg/mL, Imipenem at 1 mg/mL, Methicillin at 1 mg/mL, and Ampicillin at 10 mg/mL. All extracts were tested at maximum concentration of 10 mg/mL.

## Data Availability

Data is contained within the article.
